# Evidence to service gap: cardiac rehabilitation and secondary prevention in rural and remote Western Australia

**DOI:** 10.1186/s12913-018-2873-8

**Published:** 2018-01-30

**Authors:** Sandra Hamilton, Belynda Mills, Shelley McRae, Sandra Thompson

**Affiliations:** 1 Western Australian Centre for Rural Health, University of Western Australia, 35 Stirling Highway, Crawley, WA 6009 Australia; 2 National Heart Foundation of Australia, 334 Rokeby Road, Subiaco, WA 6009 Australia; 3 Western Australian Centre for Rural Health, PO Box 109, Geraldton, WA 6531 Australia

**Keywords:** Cardiac rehabilitation, Secondary prevention, Rural and remote, Western Australia, Access, Health services, Alternative methods

## Abstract

**Background:**

Cardiovascular disease (CVD), a leading cause of morbidity and mortality, has similar incidence in metropolitan and rural areas but poorer cardiovascular outcomes for residents living in rural and remote Australia. Cardiac Rehabilitation (CR) is an evidence-based intervention that helps reduce subsequent cardiovascular events and rehospitalisation. Unfortunately CR attendance rates are as low as 10–30% with rural/remote populations under-represented. This in-depth assessment investigated the provision of CR and secondary prevention services in Western Australia (WA) with a focus on rural and remote populations.

**Methods:**

CR and Aboriginal Community Controlled Health Services were identified through the *Directory of Western Australian Cardiac Rehabilitation and Secondary Prevention Services 2012.* Structured interviews with CR coordinators included questions specific to program delivery, content, referral and attendance.

**Results:**

Of the 38 CR services identified, 23 (61%) were located in rural (*n* = 11, 29%) and remote (*n* = 12, 32%) regions. Interviews with coordinators from 34 CR services (10 rural, 12 remote, 12 metropolitan) found 77% of rural/remote services were hospital-based, with no service providing a comprehensive home-based or alternative method of program delivery. The majority of rural (60%) and remote (80%) services provided CR through chronic condition exercise programs compared with 17% of metropolitan services; only 27% of rural/remote programs provided education classes. Rural/remote coordinators were overwhelmingly physiotherapists, and only 50% of rural and 33% of remote programs had face-to-face access to multidisciplinary support. Patient referral and attendance rates differed greatly across WA and referrals to rural/remote services generally numbered less than 5 per month. Program evaluation was reported by 33% of rural/remote coordinators.

**Conclusion:**

Geography, population density and service availability limits patient access to CR services in rural/remote WA. Current inadequacies in delivering comprehensive centre-based CR in rural/remote settings impedes management of cardiovascular risk and opportunities for event reduction. Health pathways that ensure referral and continuity of care are needed, with emerging technology-based CR support to supplement centre-based CR services requiring assessment. Implementing systematic data collection across services to establish benchmarks and enable service monitoring and evaluation is needed.

## Background

Cardiovascular disease (CVD) is a leading cause of morbidity and mortality in Australia and the leading disease category for health-care expenditure [1, 2]. In 2011, CVD accounted for 31% of all Australian deaths, with coronary heart disease (CHD) accounting for 15% of all deaths and 47% of CVD deaths, many premature and preventable [3].

Although metropolitan and rural levels of cardiovascular disease (CVD) are similar, cardiovascular outcomes for rural and remote people are poorer [4]. Rural and especially remote regions of Australia have a substantially higher proportion of Aboriginal and Torres Strait Islander (hereafter Aboriginal) people [5], a group with higher rates and earlier onset of CVD compared with non-Aboriginal people [6, 7]. Vulnerable patient populations and those with multiple comorbidities are less likely to receive, adhere to and complete cardiac rehabilitation [8, 9], leading to suboptimal secondary prevention and clinical benefit.

Cardiac rehabilitation (CR) is a coordinated multidimensional evidence-based strategy that aims to assist patients with CVD return to “an active and satisfying life and to prevent the recurrence of cardiac events” [10]. Secondary prevention of CVD (hereafter referred to as secondary prevention) is defined as “healthcare designed to prevent recurrence of cardiovascular events or complications of CVD in patients diagnosed with CVD” [11]. Although these definitions are similar, CR may be time limited, whereas secondary prevention proposes a cardiac rehabilitation continuum where care is provided for the rest of a person’s life according to need [11].

CR results in improved clinical and behavioural outcomes for patients with CVD [8, 12, 13]. Clinical benefits include improved cardiovascular risk factor profile and symptoms, better adherence to pharmacotherapy, improved exercise tolerance, a reduction in readmissions for myocardial infarction or revascularisation procedures, and a 25% reduction in mortality [8, 13, 14]. Improved psychosocial wellbeing and quality of life [13], reduced levels of stress, depression and cigarette smoking have also been demonstrated [8, 14, 15]. In Western Australia (WA), more than 40% of CHD events, 50% of CHD death and 35% of nonfatal myocardial infarctions occur in people with existing CHD [16].

Unfortunately, attendance rates at traditional CR programs, both globally and in Australia, are estimated to be as low as 10–30% with rural, remote and Aboriginal populations under-represented [8, 10, 12]. Low attendance rates reflects both healthcare service-related factors (health professionals and health care service and systems) and patient-related barriers to attendance or adherence despite referral [17–21]. Barriers and contributory factors are documented in Table 1.

**Table 1:** Barriers to cardiac rehabilitation

	Barrier	Contributory factor
Healthcare Professional / Health Service related Referral and access	Referral failure	Clinician awareness and recommendation, inadequate referral pathways, lack of a dedicated CR coordinator, vulnerable patient populations.
	Program inflexibility	Accessibility, inflexible program hours, failure to meet individual needs, geographical location (distance, time and transport difficulties).
	Program availability	Absence of local program, limited program places, fragmented funding, geographical location.
Patient related	Access to services:	Transport, work, social/family, distance, financial, geographical
	Patient perception	Need, value, knowledge, futility, views of health system, financial considerations
	Lack of motivation, or commitment or adherence	Lack of energy, functional impairment, vulnerable patients, smoking
	Vulnerable patient populations:	Aboriginal, other ethnic minority populations, women, rural patients, older patients, comorbidities, socio-economically disadvantaged
	Lack of support	Health Professionals, family, social
	Type of cardiac event	Patients following AMI and PCI less likely to attend than CABG

Key: *AMI* acute myocardial infarction, *PCI* percutaneous intervention, *CABG* coronary artery bypass graft

Barriers to CR attendance for rural, remote and Aboriginal patients are similar but of greater magnitude [18, 22]. A cross-sectional survey of Aboriginal cardiovascular patients and Aboriginal Health Workers in remote Queensland identified large family size and family support, post-surgical pain, lack of knowledge about CR, and low income as barriers to CR attendance [22]. A Canadian study examined CR barriers by rurality and socioeconomic status and demonstrated that rural patients attended significantly fewer CR sessions and had significantly greater barriers compared to urban residents: the most prominent barriers were distance, cost and transportation issues [18]. Brual et al. assessed the effect of drive time on CR service utilization and found that a drive time of 60 min or more influenced patient referral and enrolment [23].

Given the service and patient related barriers to traditional CR, a diverse array of alternative models of CR have been developed, including multifactorial patient-centred telehealth and community- or home-based CR, and can be preferred by many patients [15, 24]. These programs offer flexible and individualised management of CVD through in-person visits, community programs and home manuals with mobile support via phone or other electronic means [25]. These have produced similar reductions in CVD risk factors compared with traditional outpatient CR [15]. Following a 2004 review of the evidence for non-conventional CR models of care, Dollard et al., concluded that home-based CR had the greatest potential for improving accessibility to CR for rural and remote patients [26]. However, there remains a paucity of evidence for the adaptability and effectiveness of these programs in rural, remote and Aboriginal patients [15].

This in-depth assessment mapped CR and secondary prevention services in WA to inform future work investigating the processes and systems of referral and the implementation of alternative methods of CR delivery in rural and remote settings.

## Methods

### Study design

A mixed methods in-depth study of CR services in WA was undertaken from March to July 2015 and included the following agencies: North and South Metropolitan Health Services, West Australian Country Health Service (hospital and community care programs); private physiotherapy providers; and primary care including Aboriginal Community Controlled Health Organisations (ACCHOs).

A structured interview guide was developed by the first author in consultation with the co-authors and piloted with a local CR coordinator before use. Quantitative and open-ended interview questions (Table 2) were based on a previous survey which examined CR and secondary prevention for Aboriginal people post discharge for Acute Coronary Syndrome [27]. However, this was broadened and adapted to incorporate main elements for inpatient and outpatient CR as documented in the National Heart Foundation of Australia and the Australian Cardiac Rehabilitation Association Recommended Framework for Cardiac Rehabilitation 04 [10]. Quantitative data was based on documentation of CR coordinator reports at interview, with a summary of service data forwarded by email wherever possible.

**Table 2:** Components of cardiac rehabilitation included in the interview guide

Component	Content
Core component of cardiac rehabilitation	*Inpatient CR:* Type of program; type of facility; main elements of inpatient CR (basic information and reassurance, supportive counselling, mobilization and resumption of activities, discharge planning, and referral to outpatient CR and ongoing care). *Outpatient CR:* Pre-cardiovascular surgery review; assessment, review and follow-up; low or moderate intensity physical activity; education, discussion and counselling; ongoing assessment and management, and monitoring and evaluation.
Program information	Type of health service; type of program delivery; patient eligibility; program duration and frequency; attendance fee; patients from diverse populations; and patient monitoring and referral for further medical review.
Referral and attendance	Patient referral pathway; patient attendance and completion numbers; patient outcomes; and exit strategies for patients on completion.
Program coordination and multidisciplinary team	Dedicated program coordinator; multidisciplinary team availability; use of cardiac rehabilitation guidelines; sustainability of program and program and service contact details.

Key: *CR* Cardiac Rehabilitation

Cardiac rehabilitation services and program coordinators were identified through the Heart Foundation (HF) *Directory of Western Australian Cardiac Rehabilitation and Secondary Prevention Services 2012* [28]. This directory provides a comprehensive list of state-wide CR services in WA. Identified services were contacted and coordinators invited to participate, given verbal and written information about the study. Interviews were scheduled at the coordinator’s convenience following informed consent. Interview responses were documented and digitally recorded with the coordinator’s consent.

The Western Australian Country Health Service and University of Western Australia Human Ethics Committees approved the study.

### Data analysis

Quantitative data was analysed using IBM SPSS Statistics 22 with descriptive statistics to report both continuous and categorical data. Qualitative data was analysed manually to identify program content, concepts and themes in relation to the research aims. The Australian Government Department of Health’s Rural, Remote and Metropolitan Areas (RRMA) classification was utilised to classify service location. The RRMA classification is based on Statistical Local Areas (SLA) and based primarily on population numbers and an index of remoteness (Table 3) [29].

**Table 3:** Structure of the Rural, Remote and Metropolitan Areas (RRMA) classification [29]

Zone		Class
Metropolitan zone	M1	Capital cities
	M2	Other metropolitan centres (urban centre population ≥ 100,000)
Rural zone	R1	Large rural centres (urban centre population 25,000–99,999)
	R2	Small rural centres (urban centre populations 10,000–24,999)
	R3	Other rural centres (urban centre populations < 10,000)
Remote zone	Rem1	Remote centres (urban centre population ≥ 5000)
	Rem2	Other remote centres (urban centre population < 5000)

## Results

In total 70 services were identified from the CR Directory, including 18 ACCHOs. Of these, 38 services provided a CR/secondary prevention program, 12 had discontinued CR programs and 14 ACCHOs did not offer CR or heart health programs. Difficulty in establishing contact meant it is unknown for 6 services what CR if any they offer (Table 4). Thirty four Cardiac Rehabilitation Coordinators were interviewed from March to July 2015: four who were contacted declined interview due to work load constraints.

**Table 4:** Identified programs using the Heart Foundation Directory 2012

	Overall *n*	Rural *n* (%)	Remote *n* (%)	Metropolitan *n* (%)
All services listed in Heart Foundation Directory				
*Total*	*70*	*16 (23)*	*30 (43)*	*24 (34)*
No Program	14	1 (7)	13 (93)	0
Program Discontinued	12	3 (25)	2(17)	7 (58)
Unknown	6	1 (17)	3 (50)	2 (33)
Program offered	38	11 (29)	12 (32)	15(39)
Interviewed	34	10 (30)	12 (35)	12(35)
Aboriginal Medical Services				
*Total*	*18*	*2 (11)*	*15 (83)*	*1 (6)*
Program offered	2	1 (50)	0 (0)	1(50)
No Program	14	1 (7)	13 (93)	0 (0)
Unknown	2	0 (0)	2 (100)	0 (0)
Interviewed	2	1 (1)	0 (0)	1 (1)

### Geographical location of services

From the 34 services where a representative was interviewed, 22 (65%) were located in larger rural and remote towns or regional cities. The remaining 12 (35%) services were located in metropolitan Perth (Table 5). Of the 22 rural and remote programs, 11 (50%) were located in the health regions of southern WA: the Wheatbelt, South West, and Great Southern. The remaining 50% were based in the Kimberley (*n* = 3), Pilbara (*n* = 3), Midwest (*n* = 3), and Goldfields (*n* = 2), an area of some 2,312,237 km^2^ or 87% of the total WA land mass.

**Table 5:** Cardiac rehabilitation service provision in Western Australia

	Rural *n* (%)	Remote *n* (%)	Metropolitan *n* (%)
CR service location	10 (30)	12 (35)	12 (35)
Public inpatient CR service	3 (30)	1 (8)	3 (25)
Private inpatient CR service	0	0	1 (8)
Public hospital-based outpatient CR service	8 (80)	9 (75)	6 (50)
Public community-based outpatient CR service	2 (20)	3 (25)	5 (42)
Private hospital and community-based CR service	0	0	1 (8)
Supervised exercise classes	10 (100)	11 (92)	12 (100)
Comprehensive education classes	3 (30)	2 (17)	7 (58)
Generic chronic condition rehabilitation classes	6 (60)	10 (80)	2 (17)
Multidisciplinary CR team care	5 (50)	4 (33)	8 (67)
CR program evaluation	4 (40)	4 (33)	9 (75)
CR guideline use	6 (60)	8 (67)	12 (100)

### Inpatient cardiac rehabilitation service provision

Eight of 34 coordinators reported responsibilities for inpatient CR (Table 5); all programs offered individualised patient-centred inpatient CR. Metropolitan programs were more likely to offer all the main elements of inpatient CR (Table 2) than rural and remote programs.

### Outpatient cardiac rehabilitation service provision

All 34 services provided outpatient CR, 68% hospital-based, 29% community-based and one private provider was both hospital- and community-based (Table 5). No coordinator offered comprehensive home-based or an alternative method of program delivery. A minority (18%) of coordinators reported offering a home program, home exercise, or a graduated walking program; in one instance, this was for patients who did not want to attend group sessions. Other methods of support included telephone support, home visits if required (metropolitan private service and rural and remote programs) with two CR coordinators in remote settings reporting visits to Aboriginal communities.

Program length varied from 6 to 24 weeks, with some services offering an ongoing program. Rural and remote programs (45%) were more likely to have an ongoing access policy compared with 25% of metropolitan programs. Most classes (exercise or education) were run on set days and at set times. Flexibility if available occurred through an option of days or extended hours on a set days. One program reported an after-hours education class which improved attendance. Where comprehensive programs were delivered, these included exercise (low or moderate intensity physical activity), education, psychosocial support, self-management support, and management of symptoms and medications. Most programs offered supervised exercise classes at a low and/or moderate intensity; one remote nursing post offered telephone liaison, support and education. Many programs were unable to offer formal education classes and in these instances informal education was given at all exercise classes. Programs in rural and remote settings were more likely to be generic chronic condition rehabilitation compared with metropolitan programs (Table 5).

Table 6 shows the program delivery characteristics of program coordinators who were generally physiotherapists or registered nurses and more likely to be female (75%) regardless of rurality. All rural and 83% of remote program coordinators were physiotherapists; CR coordinators in rural and remote programs had only a part time CR role generally as only one component of the person’s overall role. The length of time of coordinators had worked in their role varied, with a wide range in all program settings. Multidisciplinary CR team care was more likely in metropolitan programs (Table 5), with rural and remote programs having the most challenges accessing multidisciplinary input which often had to occur by telephone.

**Table 6:** Program delivery characteristics of cardiac rehabilitation coordinators

	Overall *n* (%)	Rural *n* (%)	Remote *n* (%)	Metropolitan *n* (%)
Health discipline of CR Coordinator				
Registered nurse	8 (24)		2 (17)	6 (50)
Physiotherapist	23 (67)	10 (100)	10 (83)	3 (25)
Exercise Physiologist	2 (6)			2 (17)
Health Promotion officer	1 (3)			1 (8)
Full time equivalent (FTE) in CR role				
≤ 0.8 FTE (range 0.2–0.8)	27 (79)	10 (100)	12 (100)	5 (42)
1.0 FTE	7 (21)	0 (0)	0 (0)	7 (58)
Months in position				
mean ± SD	52.8 ± 67.7	65.1 ± 97.2	48.5 ± 58.7	47.8 ± 53.4
Range (min-max)	1–276	3–276	1–192	3–192

### Referral and attendance

Western Australia (and Australia) has no standard system of CR data collection so our quantitative analysis is limited, based on coordinator reports rather than analysis of consistent, routinely collected service data. All program coordinators reported receiving patient referrals from metropolitan public hospitals. Table 7 shows other referral sources. Cardiac rehabilitation coordinators, nursing and allied health staff and cardiologists were most likely to make the referral and the most common method of referral was a referral letter or a copy of the discharge letter. Other methods of referral included telephone calls, facsimile, eReferral and email. The timeliness to receipt of referral following discharge varied, with 46% of referrals received at the time of discharge with others following in the ensuing weeks [1–2 weeks (15%), 3–4 weeks (18%), 1–3 months (12%) and other (9%)].

**Table 7:** Referral source

	% of CR Coordinators reporting referral source
Metropolitan public hospitals	100
Regional public hospitals	50
Metropolitan private hospitals	60
Regional private hospitals	35
General practice	60
Aboriginal Medical Services	50
Private cardiology rooms	3
Self-referrals	40
Aboriginal community clinics.	17

Referral numbers varied widely and were greater for metropolitan services. The majority of rural and remote services received less than 5 cardiac referrals per month, with some remote services receiving less than 5 per year. In comparison, referral rates at metropolitan services varied from 2 to 105 per month, with higher referral rates at tertiary hospitals, a private cardiovascular prevention and rehabilitation service (73–105 per month) and a larger metropolitan community service (47 per month). Heart Failure services and smaller community services received 2 to 27 referrals per month.

The percentage of those patients who were referred to CR and then attended was reported by 70% of the CR Coordinators. Attendance rates were similar across regions (Table 8). Two thirds of patients who attended CR were male (Table 8). Limited information was available for patient completion rates with only 35% of coordinators (*n* = 12) reporting data. Of these, 9 coordinators reported completion rates (defined as completing > 75% of program sessions) of 75–100%.

**Table 8:** Percent attendance of referred patients

	Rural			Remote			Metropolitan		
	Mean %	Range %	SD	Mean %	Range %	SD	Mean %	Range %	SD
Referred patients who attended	75	33–100	29.2	75	50–100	22.9	72	30–100	29.7
	%			%			%		
Male	60			66			60		

### Data collection, documentation and evaluation

All coordinators reported conducting biomedical and exercise assessments, monitoring progress and documenting findings and patient progress. However, no standard system of data collection was identified. The most common (65%) method of documentation was reporting the patient’s progress in their medical record. Other methods of documentation included: assessment sheets (*n* = 3); nursing notes (*n* = 1); traditional physiotherapy notes (*n* = 2); SF36 form (*n* = 2); an attendance record (*n* = 4); own data base (*n* = 3); Cardiobase (*n* = 2); completion letters (*n* = 2) and a check list at group sessions (*n* = 1). Two programs (CR and Heart Failure) at one metropolitan tertiary hospital used multiple methods of the data documentation described above.

A third of coordinators reported undertaking patient evaluation which was described as administering a patient evaluation form (25%) or a patient satisfaction survey (8%). Program evaluation was reported by 50% of coordinators and included quarterly or yearly reports to a funding body or management, evaluation of improvement in patient risk factors and outcome measures and evaluation of education classes. Evaluation frequency was variable and was reported to occur quarterly, 6 monthly, yearly to two yearly depending on service requirements.

### Cardiac rehabilitation guideline use

Overall, 77% of coordinators reported that their program was based on CR guidelines (Table 5). The National Heart Foundation of Australia and Australian Cardiac Rehabilitation Association *Recommended Framework for Cardiac Rehabilitation ‘04* [10] was the most frequently utilised guideline. Table 9 documents other guideline utilization. A minority (9%) of coordinators were unsure of the guidelines utilised and 6% stated that their program was “not really” guideline based.

**Table 9:** Cardiac rehabilitation guideline use

	% of CR Coordinators reporting use
National Heart Foundation of Australia and Australian Cardiac Rehabilitation Association *Recommended Framework for Cardiac Rehabilitation ‘04* [10]	60
The Australian Cardiovascular Health and Rehabilitation Association *Core Components of Cardiovascular Disease Secondary Prevention and Cardiac Rehabilitation 2014* [30]	15
Department of Health, Western Australia, *Cardiovascular rehabilitation and secondary prevention pathway principles for Western Australia, 2014* [32]	15
National Health and Medical Research Council, *Strengthening Cardiac Rehabilitation and Secondary Prevention for Aboriginal and Torres Strait Islander Peoples: A Guide for Health Professionals* [38].	0
Utilization of more than one guideline, including WA Models of Care and interstate or international programs/guidelines	20

## Discussion

The recommended core components of cardiovascular disease secondary prevention and cardiac rehabilitation in Australia suggest that it is essential for all CR services to provide the most comprehensive service possible within resource availability [30]. This in-depth assessment of CR services in WA found a service gap in delivery of comprehensive CR and secondary prevention for people residing in rural and remote settings, reflecting limitations related to geographical location, service design and comprehensiveness, health professional availability, referral pathways and numbers. Data collection is inconsistent and given inadequate attention, limiting program evaluation and the potential for improvements in quality.

The delivery of comprehensive CR programs in rural and remote WA is challenged by geographical location, low population density and limited service availability. Sixteen percent of the Australian population live in geographical regions with poor or no access to CR and secondary prevention services [31]. Distance, transport difficulties, cost and family responsibilities are significantly greater barriers to CR access for rural compared with metropolitan patients [18]. Most CR services in rural/remote WA are located in regional centres, creating access challenges related to transport, time and cost for residents outside these centres. We have demonstrated lower CR referral rates in rural and remote WA compared with metropolitan rates, in part reflecting a lower population density. Referral failure is a known barrier to CR enrolment and attendance [17–20], however, Brual et al. report that a drive time of greater than 60 min effects CR referral and enrolment but not attendance once enrolled [23]. This highlights the importance of effective referral systems, pathways and offering alternative means of CR service delivery for cardiac patients at greater geographic disadvantage.

Traditional service delivery for CR is centre-based and ideally it incorporates a comprehensive program of assessment, education and self-management strategies to promote behaviour change, exercise, psychosocial support, medical follow-up and service evaluation and quality improvement [25, 30, 32]. However, alternative models of CR utilising patient-centred telehealth and technology (internet and mobile health via smartphone apps) and supporting home-based CR have been developed and are preferred by many patients in all geographical settings [15, 24, 33, 34]. Despite these advances, in rural/remote WA the majority of CR services were centre-based exercise classes with informal education or chronic condition rehabilitation classes that accommodates the cardiac patient. A fifth of coordinators reported the capacity to provide support for home exercise but there was no comprehensive home-based or alternative method of program delivery provided. Efforts to utilise more modern approaches based on advances in technology to delivery of CR in rural and remote WA are needed. Mobile health technology for the delivery of a comprehensive CR program demonstrated improved uptake and completion rates and improved health outcomes equal to centre-based CR in a Brisbane study [35, 36]. Implementation of such technology has great potential for overcoming the challenges to service delivery associated with geographical location and isolation. However, its benefits are still to be tested in rural and remote settings and its utility to support Indigenous people remains to be assessed.

Given the complexity of a cardiac patient’s recovery, CR is ideally delivered by a multidisciplinary team with appropriate qualifications and expertise [30]. Less than half of rural/remote services reported the capacity to provide face-to-face multidisciplinary team care, so that access to multidisciplinary expertise was often by telephone. In rural and remote programs, coordination of CR was only one component of multiple roles for those who were designated coordinators (90% of whom were physiotherapists) and this, together with the centre-based nature of the program and limited access to a multidisciplinary team, limited program comprehensiveness and flexibility.

Thirty percent of services listed in the Heart Foundation Directory either did not provide CR or the program had been discontinued, suggesting considerable service turnover. All 14 services that were unable to provide a CR program were rural and remote ACCHOs. Although the reasons for this is unclear, it possibly relates to multiple factors such as geographical context, population density, provision of CR by the Country Health Service in some locations, ACCHO funding cycles and staff recruitment and retention. Program discontinuation in rural and remote regions in two instances (and possibly a further two) was related to the “rationalisation” of the service to an outreach physiotherapy service on a once per month basis. One rural private physiotherapy clinic discontinued CR. Discontinuation of metropolitan services related to withdrawal of telephone CR coaching and restructuring of CR services with the opening of a new tertiary hospital.

Ongoing attention to improvement of CR services and the provision of flexible, accessible and guideline-based alternative methods of CR delivery in rural areas is needed in order to improve access to CR for rural and remote patients in WA. Accessibility requires that all dimensions of access as described by Levesque et al., are considered and encompass approachability, acceptability, availability and accommodation, affordability and appropriateness (including cultural appropriateness [37]. The relatively high level of completion of CR described by coordinators indicates that an essential step in CR delivery is referral and initial attendance. Endorsement of guideline implementation, and a structured integrated health pathway to ensure coordinated referral and management of CR and secondary prevention are essential. Raising awareness and quality professional development for all health professionals involved in supporting CVD patient journeys could improve knowledge and use of CR guidelines.

### Strengths and limitations

The strength of our study relates to the in-depth assessment undertaken, the rich level of data obtained from the CR coordinators and high participation of services in the study. While the Heart Foundation (HF) *Directory of Western Australian Cardiac Rehabilitation and Secondary Prevention Services 2012* is a comprehensive directory of CR services and a small number of new services were known and contacted, it remains possible that a small number of services were not identified. A further limitation is the inability to estimate the number of patients who are not referred to CR, as access to concurrent discharge data for the number of eligible patients was not included in the study.

CR services in WA currently lack a standard method of data collection, limiting availability and analysis of quantitative and qualitative client data. A variety of data collection and evaluation methods were used by services across WA, so data available differed across services, and was particularly deficient for those in rural and remote areas. Inconsistency of quantitative data collection across services precluded comparisons between regions and comparative statistical analysis. The inconsistency of data collection for referral, attendance and completion rates also prevented a per capita comparison between rural, remote and metropolitan services. Population density is lower in rural and remote regions leading to lower referral numbers, however, most rural and remote CR services were situated in larger towns or regional cities. This means those living outside of regional centres have longer travel times and costs in order to attend, with time and distance being a strong influence on referral and enrolment rates [23]. Australian guidelines recommend the collection of information to identify problem areas and measure change [30, 38]. Implementing a minimum dataset to record and monitor CR and secondary prevention in WA [11, 32] would enable evaluation of the quality and outcome of participation in each of the five core components of CR [30]. A nationally agreed minimum data set, if implemented at a national level, could enable service performance indicators for all phases of CR; tracking patient referral, attendance, adherence, completion rates and outcomes. Establishing consistent relevant benchmarks will help ensure greater accountability for service function in rural/remote settings.

## Conclusions

Geography, population density and service availability limits patient access to CR services in rural/remote WA. Current inadequacies in comprehensive centre-based CR in rural/remote settings impedes management of cardiovascular risk and opportunities for event reduction. Health pathways that ensure referral and continuity of care are recommended, with implementation and evaluation of emerging technology-based CR support to add value to centre-based CR services. Implementing systematic data collection across services to establish benchmarks and enable service monitoring and evaluation is an essential component of progress. Future work is required by linking discharge data to assess and improve the proportion of eligible patients referred to CR and so that attention is given to addressing disparities between metropolitan, rural and remote settings.

## Abbreviations

ACCHO: Aboriginal community controlled health organisation
CHD: Coronary heart disease
CR: Cardiac rehabilitation
CVD: Cardiovascular disease
WA: Western Australia
